# Procalcitonin level as a marker of severe sepsis and septic shock patients who required polymyxin-B immobilized fiber with direct hemoperfusion

**DOI:** 10.1186/cc11692

**Published:** 2012-11-14

**Authors:** T Ikeda, K Ikeda, S Suda

**Affiliations:** 1 Tokyo Medical University Hachioji Medical Center, Tokyo, Japan

## Background

When septic patients progress to endotoxin shock, the mortality rate becomes high and is within 30 to 80% in those with multiple organ failure.

## Methods

We evaluated the levels of procalcitonin (PCT) and markers of sepsis (IL-6, IL-8, IL-1ra and PAI-1) in 159 patients who had severe sepsis and septic shock. Before starting the polymyxin-B immobilized fiber with direct hemoperfusion (PMX-DHP) treatment, patients were divided into three groups according to their PCT levels (L group: 0.5 <PCT ≦2.0 ng/ml, M group: 2.0 <PCT ≦10.0 g/ml, H group: PCT >10.0 ng/ml).

## Results

Sixty-two percent of the patients showed high PCT levels (>10.0 ng/ml). The APACHE II score and Sequential Organ Failure Assessment score tended to be high in the H group, but there was no significant difference among the groups. The survival rate declined with high PCT levels. The levels of inflammatory (IL-6 and IL-8) and anti-inflammatory (IL-1ra) cytokines tended to be high in the H group, but there was no statistically significant difference among the groups. On the other hand, the PAI-1 level was significantly increased in the H group (498 ± 499 ng/ml) compared with the L group (157 ± 103 ng/ml) and M group (311 ± 319 ng/ml).

## Conclusion

Sixty-two per cent of patients who required PMX-DHP treatment had high PCT levels. PCT might be a mediator of sepsis and sepsis markers. PCT can potentially affect sepsis-related markers.

